# Communication about breast cancer genetic counseling with patients with limited health literacy or a migrant background: evaluation of a training program for healthcare professionals

**DOI:** 10.1007/s12687-020-00497-x

**Published:** 2020-12-15

**Authors:** Jeanine van der Giessen, Mirjam P. Fransen, Peter Spreeuwenberg, Mary Velthuizen, Sandra van Dulmen, Margreet G. E. M. Ausems

**Affiliations:** 1 Division Laboratories, Pharmacy and Biomedical Genetics, Department of Genetics, University Medical Center Utrecht, P. O. Box 85500, 3508 GA Utrecht, The Netherlands; 2 Department of Public and Occupational Health, Amsterdam Public Health Research Institute, Amsterdam UMC, University of Amsterdam, P. O. Box 22660, 1100 DD Amsterdam, The Netherlands; 3 NIVEL (Netherlands Institute for Health Services Research), P. O Box 1568, 3500 BN Utrecht, The Netherlands; 4 Radboud Institute for Health Sciences, Department of Primary and Community Care, Radboud University Medical Center, P. O Box 9101, 6500 HB Nijmegen, The Netherlands

**Keywords:** Breast cancer, Referral, Genetic testing, Health literacy, Blended training program

## Abstract

Access to breast cancer genetic counseling is suboptimal for patients with limited health literacy or a migrant background due to ineffective communication and lack of healthcare professionals’ recommendation. This study examines the effect of a blended training program (Erfo4all) for healthcare professionals on their awareness, knowledge, and self-efficacy towards communication about genetic counseling with patients with limited health literacy or a migrant background. In total, 59 breast surgeons and specialized nurses from 16 Dutch hospitals completed an online module and group training. Knowledge, self-assessed awareness, and self-efficacy were assessed before the training and 33 participants also completed a posttest questionnaire 6 months after the training program. We also assessed the perceived applicability and relevance of the training program from healthcare professionals’ perspectives. We found a significant increase in self-assessed awareness of the prevalence and impact of limited health literacy and in healthcare professionals’ self-efficacy to recognize limited health literacy and to communicate effectively with patients with limited health literacy or a migrant background. We did not find an increase in knowledge score. Almost all healthcare professionals reported that they use the techniques learned in the training, such as the teach-back method and plain language, and felt more confident discussing breast cancer genetic counseling. Our results suggest that a blended training program for healthcare professionals has potential to improve their ability to communicate effectively about breast cancer genetic counseling with patients with limited health literacy or a migrant background and offers a promising way to increase the referral rate for these groups of patients.

## Introduction

Breast cancer, the most commonly diagnosed type of cancer, affects about 12% of women worldwide (Bray et al. 2018). Between 5 and 10% of breast cancers are associated with a hereditary predisposition. Pathogenic variants in the BRCA1 and BRCA2 genes are associated with about 20% of familial breast and ovarian cancers (Gorodetska et al. 2019). Female breast cancer patients with a BRCA1 or BRCA2 pathogenic variant have an increased risk of a second primary breast cancer and/or ovarian cancer. A pathogenic variant may also affect healthy family members, so genetic testing of breast cancer patients at risk of carrying a pathogenic variant is important (Byrski et al. 2014; Godet and Gilkes 2017; Wevers et al. 2012). Healthcare professionals (surgical oncologists, medical oncologists, and specialized nurses) should identify patients at risk of carrying a variant in a breast cancer gene (e.g., BRCA1, BRCA2, CHEK2, PALB2, ATM genes), inform them about genetic testing, refer them for genetic counseling, or request a test themselves. Yet, despite the fact that genetic testing has been available for over two decades, many eligible women do not receive BRCA testing (Knerr et al. 2019) and for certain groups of patients, the substantial underutilization of genetic testing is even larger (Cohen et al. 2019). Previous research has shown that there is unequal access to cancer genetic testing for patients with lower educational levels and those with a migrant background (van Riel et al. 2012; Kurian et al. 2017; Delikurt et al. 2015; McCarthy et al. 2016; van der Giessen et al. 2017). The major cause for this seems to lie in the absence of surgeon recommendations (Kurian et al. 2017; Hafertepen et al. 2017). Referral is not always adequately discussed with these groups of patients and limited health literacy as well as cultural differences seems to play a role (van Riel et al. 2012; van der Giessen et al. 2017; Baars et al. 2017; Joseph et al. 2019). This might be related to ineffective communication which is widely recognized as a major contributor to health disparities (Joseph et al. 2019). Health literacy refers to the skills to get access to, understand, appraise, and use health-related information in various domains (Sorensen et al. 2012). Estimations of the prevalence of limited health literacy in the Netherlands range between 29 and 36% in the general population (Heijmans et al. 2018; Heijmans et al. 2019). 24.4% of Dutch population has a migrant background, from which 10.5% has a western migrant background and 13.9% has a non-western background (Statistics Netherlands 2020). For effective referral, it is important that surgeons and specialized nurses, the main referrers to genetic counseling for patients with breast cancer (van Riel et al. 2012; Agnese and Pollock 2016), possess adequate awareness, knowledge, and skills to identify patients with limited health literacy (Nielsen-Bohlman et al. 2004).

Healthcare professionals seem to be insufficiently aware of the negative impact of limited health literacy on medical communication, failing to recognize limited health literacy in patients and lacking the skills to effectively discuss referral to breast cancer genetic counseling (Lea et al. 2011; Coelho 2018). They might benefit from a training in recognizing these patients and in discussing referral to genetic counseling adequately in order to optimize access to genetic care for these groups of patients. As limited health literacy, cultural factors, and language proficiency are interrelated, training programs should attend to literacy problems as well as to cultural differences (Andrulis and Brach 2007; Lie et al. 2012).

### Training program for healthcare professionals

We developed a blended training program (Erfo4all) based on healthcare professionals’ and patients’ needs and preferences, to increase healthcare professionals’ knowledge, awareness, and self-efficacy when communicating about breast cancer genetic counseling with communication-vulnerable patients, i.e., patients with limited health literacy or a migrant background (van der Giessen et al. 2020).

In our blended training program, we selected an integrated approach to health literacy and communication with patients with a migrant background. The training program was developed within the Erfo4all project, a project that aims to achieve equal access to breast cancer genetic counseling for all eligible patients. The program consisted of two successive parts: an online knowledge module (20 min) and a group training in one of the participating hospitals (2 h). The online module included information about the prevalence of low literacy and limited health literacy in the Netherlands, the relevance of health literacy in understanding and appraising information from healthcare professionals, and the way health literacy relates to socio-economic and background characteristics of patients. It also included information about how to communicate effectively with patients with a migrant background. In the group training, the teach-back method—a methodology used by healthcare providers to check whether a patient understands what has been discussed—was used as a technique to identify patients with limited health literacy and role-play was used as a strategy to acquire required communication skills and to practice the use of plain language (Nestel and Tierney 2007; Klingbeil and Gibson 2018; Sudore and Schillinger 2009). To further enhance healthcare professionals’ cultural competences, cultural sensitivity training techniques were introduced. The training was led by a professional trainer from Pharos (Dutch Centre of Expertise on Health Disparities) and role-play sessions, based on real life cases, were practiced with a training actress.

The aims of the current study were to assess:

I. The extent to which this training program increased:

- Healthcare professionals’ knowledge about the impact and prevalence of limited health literacy, awareness of the impact of limited health literacy on medical communication, their self-efficacy to recognize patients with limited health literacy and to communicate effectively about breast cancer genetic counseling
- Healthcare professionals’ awareness of the importance of taking into account cultural factors when communicating with patients with a migrant background and their self-efficacy in coping with these cultural factors and with language barriers

II. The perceived applicability and relevance of the training program from healthcare professionals’ perspective

## Methods

A total of 73 healthcare professionals involved in breast cancer treatment from 19 hospitals in the Netherlands responded to an invitation to participate in the Erfo4all training program. Before the start of the training program, they were asked to fill out an initial online questionnaire (T0) to get access to the online knowledge module. Within 2 weeks, they were invited to attend a group training on location. We used a pre-/posttest design to evaluate the effect of the Erfo4all intervention on knowledge, self-assessed awareness, and self-efficacy related to communication about breast cancer genetic counseling with patients with limited health literacy or a migrant background. Six months after the group training, the healthcare professionals were asked to fill out a second questionnaire (T1), again to assess knowledge, awareness, and self-efficacy. At T1, we added self-report questions concerning the applicability and relevance of the training program as well as healthcare professionals’ perceived awareness regarding the problem of health literacy in general and their confidence to adapt their communication effectively. Only healthcare professionals who completed the whole program and filled out the T0 and T1 questionnaire were considered in the pre- and post-intervention analysis. For the additional questions, all healthcare professionals who filled in the T1 questionnaire were included.

### Outcome variables

Outcome variables were assessed with self-constructed online questionnaires, before (T0) and 6 months after the training (T1). The items in these questionnaires were based on the intervention mapping approach and the matrix of change used for the development of our training program (van der Giessen et al. 2020).

*Knowledge* was assessed with five multiple choice questions focusing on the prevalence of low literate adults in the Netherlands, limited (health) literacy among people with a migrant background, the prevalence of adults with limited health literacy in the Netherlands, the level of education in relation to the level of health literacy, and option of using of a professional interpreter (self-reported). Each item was rated as correct (1) or incorrect (0) and a total knowledge score was computed as the number of correct answers.

*Awareness* was assessed by three items: prevalence and impact of health literacy in the Netherlands, impact of health literacy on medical communication, and the importance of taking into account cultural factors when communicating with patients with a migrant background.

Items were scored on a 5-point Likert scale ranging from (1) hardly aware to (5) very highly aware.

*Self-efficacy* was assessed by five statements on having confidence in recognizing limited health literacy in patients, communicating effectively about breast cancer genetic counseling with patients with limited health literacy, understanding which customs and habits from patients with a migrant background might influence communication, coping with cultural factors in communication with patients with a migrant background, and coping with a language barrier.

Items were scored on a 5-point Likert scale ranging from (1) totally disagree to (5) totally agree.

At T1, we also assessed healthcare professionals’ perceived awareness regarding the problem of limited health literacy in general and their confidence to tailor communication about breast cancer genetic counseling to the needs of communication-vulnerable patients. These additional items were scored on a 5-point Likert scale ranging from (1) totally disagree to (5) totally agree. The relevance of the training was assessed by asking whether the trained healthcare professionals shared information about the training with colleagues in multidisciplinary oncology meetings. Application of skills, such as the use of the teach-back method and plain language, was assessed with a 5-point Likert scale ranging from (1) never to (5) very regularly.

### Statistical analysis

Univariate analysis was used to describe the background characteristics of healthcare professionals.

Categorical data, number of healthcare professionals, discipline, sex, and clinical setting are presented in numbers and percentages. Continuous data, like age and work experience, are presented as means and standard deviations. To analyze T0 and T1 data, we performed paired analysis or repeated measurement, since multiple responses from the same subject cannot be regarded as independent from each other. In this analysis, subjects were included only when data were available from both time points (T0 and T1). To check for potential selective drop-out, we compared the group that only completed the T0 (T0 only) with the group who completed both questionnaires (T0+T1) and looked for differences in demographics and outcome variables. To analyze pre-post differences in the outcome measures’ knowledge, awareness, and self-efficacy, we used the Wilcoxon signed-rank for related samples. Tests for statistical significance were two-sided with *α* = 0.05. Statistical analyses were performed using SPSS version 24.0 (SPSS Inc, Chicago, IL).

## Results

The baseline questionnaire (T0) was completed by 65 participants of whom 59 participants from 16 hospitals completed the whole training program (online module and group training). In total, 37 participants filled out the T1 questionnaire, of whom 33 participants from 14 hospitals filled out both questionnaires (T0 and T1). Table 1 shows the background characteristics of healthcare professionals who only completed the first questionnaire (*n* = 59) and of those who completed both questionnaires (*n* = 33). Based on background characteristics, no statistical differences were found between both groups, indicating that drop-out between T0 and T1 was not selective.

**Table 1:** Background characteristics of healthcare professionals

Variable		*n* = 59* T0			*n* = 33** T0–T1		
Total		*n*	%	SD	*n*	%	SD
Sex	Male	11	18.6%		7	21.2%	
	Female	48	81.4%		26	78.%	
Discipline	Breast surgeon	17	28.8%		9	27.3%	
	Specialized nurse	36	61.0%		20	60.6%	
	Medical oncologist	1	1.7%		1	3.0%	
	Physician assistant	2	3.4%		2	6.0%	
	Other	3	5.1%		1	3.0%	
Age		45.8		8.5	45.2		8.7
Work experience in breast cancer care (in years)		10.7		7.1	11.0		6.0
Clinical setting	University hospital	6	10.2%		4	12.1%	
	Community hospital	53	89.8%		29	87.8%	

*n = 59: group who completed T0 questionnaire and whole training program

**n = 33: group who completed T0 questionnaire, whole training program, and T1 questionnaire

### Pre-/posttest changes in awareness, knowledge, and self-efficacy

Table 2 shows the pre-/posttest scores of the participating healthcare professionals on self-assessed awareness, knowledge, and self-efficacy (*n* = 33). At the posttest (after the training), there was a significant positive change on six outcome measures compared to the pretest (before the training). The largest increase was observed in participants’ self-efficacy. Understanding the customs and habits of patients with a migrant background, the ability to recognize limited health literacy, to communicate effectively about breast cancer genetic counseling, and to cope with cultural factors or a language barrier significantly increased from baseline to T1. Moreover, awareness of the prevalence and impact of limited health literacy in the Netherlands increased significantly. The total knowledge score did not increase over time.

**Table 2:** Awareness, knowledge and self-efficacy score of healthcare professionals before and after the training

*n* = 33	Before training (T0)		After training (T1)		Change scores T0–T1			
	*n*	%	*n*	%	*			*p* value
Awareness					**−**	**=**	**+**	
Awareness of prevalence and impact limited health literacy in the Netherlands					1	17	15	.003*
• Low	-	-	-	-				
• Barely	1	3.0%	-	-				
• Reasonable	28	84.8%	18	54.5%				
• High	3	9.1%	13	39.4%				
• Very high	1	3.0%	2	6.1%				
Awareness of the impact of limited health literacy on medical communication					5	13	15	.052
• Low	-	-	-	-				
• Barely	3	9.1%	2	6.1%				
• Reasonable	18	54.5%	10	30.3%				
• High	10	30.3%	19	57.6%				
• Very high	2	6.1%	2	6.1%				
Awareness of the importance to take into account cultural factors					5	21	7	.644
• Low	-	-	-	-				
• Barely	1	3.0%	-	-				
• Reasonable	5	15.2%	5	15.2%				
• High	23	69.7%	24	72.7%				
• Very high	4	12.1%	4	12.1%				
Self-efficacy					**−**	**=**	**+**	
Having confidence to understand which customs and habits from patients with a migrant background might influence communication					2	9	22	.000*
• Totally disagree	1	3.0%	-	-				
• Disagree	14	42.4%	1	3.0%				
• Not agree/not disagree	14	42.4%	16	48.5%				
• Agree	3	9.1%	14	42.4%				
• Totally agree	1	3.0%	2	6.1%				
Having confidence to recognize limited health literacy in patients					4	14	15	.008*
• Totally disagree	-	-	-	-				
• Disagree	7	21.2%	1	3.0%				
• Not agree/not disagree	16	48.5%	14	42.4%				
• Agree	10	30.3%	18	54.5%				
• Totally agree	-	-	-	-				
Having confidence to communicate effectively about breast cancer genetic counseling with patients with limited health literacy					0	8	25	.000*
• Totally disagree	-	-	-	-				
• Disagree	13	39.4%	-	-				
• Not agree/not disagree	14	42.4%	8	24.2%				
• Agree	6	18.2%	24	72.7%				
• Totally agree	-	-	1	3.0%				
Having confidence to cope with cultural factors in communication with patients with a migrant background					0	15	18	.000*
• Totally disagree	-	30.3%	-	-				
• Disagree	10	39.4%	1	3.0%				
• Not agree/not disagree	13	30.3%	5	15.2%				
• Agree	10	-	27	81.8%				
• Totally agree	-	-	-					
Having confidence to cope with a language barrier					2	19	12	.024*
• Totally disagree	-	-	-	-				
• Disagree	5	15.2%	2	6.1%				
• Not agree/not disagree	12	36.4%	8	24.2%				
• Agree	16	48.5%	21	63.6%				
• Totally agree	-	-	1	3.0%				
• Missing	-	-	1	3.0%				
Knowledge					**−**	**=**	**+**	
Sum scores/total knowledge Correct answers:					10	11	12	.465
• 1	1	3.0%	-	-				
• 2	5	15.2%	3	9.1%				
• 3	11	33.3%	15	45.5%				
• 4	14	42.4%	11	33.3%				
• 5	2	6.1%	4	12.1%				

### Perceived applicability and relevance of the training program

In our prior study, we assessed acceptability and usefulness of the training program directly after the group training (van der Giessen et al. 2020). Six months after completing the training program, we assessed applicability and relevance of the training program in daily practice (*n* = 37). More than 80% (*n* = 30) of the healthcare professionals reported having used plain language to explain genetic testing; of these, almost 41% (*n* = 12) reported using it (very) regularly. Even more, 92% (*n* = 34) reported applying the teach-back method to discover whether a patient understood information and to identify limited health literacy, while 62% (*n* = 23) reported using the teach-back method (very) regularly. More than 97% (*n* = 36) of the respondents felt more aware of health literacy problems and assessed their ability to recognize patients with limited health literacy higher and 86% (*n* = 32) reported that their ability to communicate effectively with these groups of patients had improved. Most healthcare professionals expected to benefit from a booster training session after 1 year and more than 41% (*n* = 15) reported sharing their experience with the training program in multidisciplinary oncology meetings with colleagues.

## Discussion

The results of our study suggest that a blended training program for healthcare professionals (i.e., breast surgeons and specialized nurses) aimed at increasing awareness, knowledge, and self-efficacy regarding limited health literacy and communication with patients with limited health literacy or a migrant background leads to significant improvement in awareness of the prevalence and impact of limited health literacy, and self-efficacy in communicating about breast cancer genetic counseling with these groups of patients. No significant differences were found in pre- and posttest knowledge scores, on awareness of the impact of health literacy on medical communication and the importance of taking into account cultural factors when communicating with patients with a migrant background. Almost half (41%) of the breast surgeons and specialized nurses who participated in the training reported to share their experience with colleagues and almost all reported to apply the techniques taught in the training in daily practice (i.e., teach-back method and using plain language).

Healthcare professionals experience several problems in discussing genetic counseling and testing with patients with limited health literacy or a migrant background. Low awareness of the problem of limited health literacy, difficulties in recognizing limited health literacy, coping with cultural factors and a language barrier in communication with patients with a migrant background, and a lack of the skills needed to discuss referral effectively with these patients are the main problems. This is often compounded by patients’ limited health literacy which has been shown to negatively affect their ability to play an active role in their own health care, by asking questions, participating in shared decision-making, and taking initiative (Rademakers et al. 2014). Health literacy is thus not an individual issue, and making health care accessible by adapting communication to patients’ understanding and abilities is critical (Heijmans et al. 2015).

The increase in self-assessed self-efficacy 6 months after the training is promising, because this outcome variable has been found to be associated with actual communication performance (Finset et al. 2003; Baumeister et al. 2019). Self-efficacy beliefs determine whether certain behavioral change will be initiated and also influence the effort one puts forth to change a certain behavior. For breast surgeons and specialized nurses, an increase in self-efficacy when communicating with communication-vulnerable patients is a prerequisite to actually changing their communication style. The use of role-play in the group training might have contributed to this result. Based on Bandura’s theory on self-efficacy (Bandura 1977), role-play has been described as one of the most effective methods to improve self-efficacy (Baumeister et al. 2019).

Communication skills training has previously been shown to produce a significant and durable increase in the self-efficacy of healthcare professionals (Banerjee et al. 2017; Saslaw et al. 2017; Norgaard et al. 2012; Coleman and Fromer 2015). However, in these interventions, training durations varied from a 3.5-h workshop to a 10-day course, and they were based on traditional face-to-face learning. For future research, it would be interesting to discover whether short- or long-term differences exist between these different approaches.

We were surprised that we did not find an increase in knowledge score mostly because other studies on health literacy training interventions with a pre-/post survey showed significant improvements in (perceived) health literacy knowledge (Coleman and Fromer 2015; Mackert et al. 2011). Furthermore, a systematic review from Liu showed that blended learning, which combines traditional face-to-face learning and e-learning, has a large consistent positive effect on knowledge acquisition (Liu et al. 2016). We therefore expected our blended learning approach to contribute to an increase in knowledge. This discrepancy could possibly be ascribed to the fact that healthcare professionals participated in our training program on a voluntary basis. It is likely that their interest in the subject and their motivation to participate in a health literacy training program provide an explanation for the relatively high knowledge scores at baseline. Their motivation to participate also reflects the fact that we, in contrast to other studies, did not highlight a lack of awareness of health literacy at baseline and also that awareness of taking into account cultural awareness was already high, so there may be a ceiling effect (Coelho 2018).

This is one of the few studies examining the effect of a training program for breast surgeons and specialized nurses with a focus on health literacy and also, to our knowledge, the first study explicitly focusing on discussing breast cancer genetic counseling with patients with limited health literacy or a migrant background. Given the high rates of low or limited health literacy in the Netherlands (36.4% of adults) (Heijmans et al. 2018), and even more in the rest of Europe (Sorensen et al. 2015), health literacy sensitive training interventions could help healthcare professionals communicate with these vulnerable groups of patients.

The dual challenges of limited health literacy and cultural differences are likely to increase due to an expanding and increasingly diverse population (Lie et al. 2012), so the effect of an integrated approach, with a focus on limited health literacy and cultural differences in one training program, is interesting for future research.

The results however should also be examined in light of the study’s limitations. First, the response rate at T1 was relatively low and this may represent a selection of healthcare professionals who are more inclined to respond. Practical reasons for this low response rate may be related to the large time period that has passed since the training and a lack of time of the participating healthcare professionals. However, despite this low response rate, the 33 healthcare professionals who completed both questionnaires do not appear to systematically differ from the group who only filled out the first questionnaire. Therefore, the results can be considered representative, and thus, we could extrapolate the results to the whole group and the time interval between pretest and posttest was long enough to avoid a testing effect. Second, as there were no standardized questionnaires available, we used a self-constructed questionnaire, developed on the basis of a theoretical approach. Because it has not been validated, it may be subject to measurement error, and conclusions cannot be made with total confidence. Third, as it was impossible to randomly assign participants to groups, we choose a pretest-posttest design without a control group. This lack of a control group made it difficult to control for confounding variables. Finally, the use of self-reported outcome measures on awareness, self-efficacy, and applied skills indicates attitudes rather than behavior. There is a risk of social desirability bias because we did not actually observe skills in daily practice, but instead asked healthcare professionals if they (felt able to) apply certain communication skills. Although it is unknown whether the increased scores on awareness and self-efficacy in this case indeed led to sustainable changes in communication behavior in daily practice, feelings of self-efficacy have been linked previously with behavioral change (Mata et al. 2019). Future studies should also examine provider-patient communication in the consulting room as we previously did in breast cancer genetic counseling (Albada et al. 2012).

In conclusion, our study shows improvements in relevant outcome measures among a diverse group of healthcare professionals involved in surgical breast cancer care in different regions in the Netherlands. It is promising that the skills learned during the training seem applicable in daily practice, even in the long term, and that healthcare professionals reported gains in awareness and self-efficacy. We implemented the Erfo4all training program in three regions in the Netherlands in different clinical settings (academic and non-academic hospitals and among healthcare professionals from different disciplines. Our previous study showed that the acceptance and perceived usability of the program was high (van der Giessen et al. 2020). Thus, widespread implementation of the training program seems feasible, making it a promising intervention for other healthcare professionals in cancer care. As genetics and genomics become part of mainstream medicine, effective communication about genetic testing becomes even more important with the potential to either reduce or exacerbate disparities in access to genetic testing (Cheng et al. 2018).
